# KMT2B promotes the growth of renal cell carcinoma via upregulation of SNHG12 expression and promotion of CEP55 transcription

**DOI:** 10.1186/s12935-022-02607-w

**Published:** 2022-05-21

**Authors:** Jia-fu Feng, Jun Wang, Gang Xie, Yao-dong Wang, Xiao-han Li, Wen-yu Yang, Yu-wei Yang, Bin Zhang

**Affiliations:** 1 NHC Key Laboratory of Nuclear Technology Medical Transformation, Mianyang Central Hospital, School of Medicine, University of Electronic Science and Technology of China, No. 12 Changjia Lane, Jingzhong Street, Mianyang, 621000 People’s Republic of China; 2 Department of Clinical Laboratory, Mianyang Central Hospital, School of Medicine, University of Electronic Science and Technology of China, Mianyang, 621000 People’s Republic of China; 3 College of Medical Technology, Chengdu University of Traditional Chinese Medicine, Chengdu, 611137 People’s Republic of China; 4 Department of Pathology, Mianyang Central Hospital, School of Medicine, University of Electronic Science and Technology of China, Mianyang, 621000 People’s Republic of China; 5 Department of Urology Surgery, Mianyang Central Hospital, School of Medicine, University of Electronic Science and Technology of China, Mianyang, 621000 People’s Republic of China; 6 Medical Laboratory, Affiliated Hospital of Southwest Medical University, Luzhou, 646000 People’s Republic of China

**Keywords:** Renal cell carcinoma, KMT2B, Long non-coding RNA, SNHG12, E2F1, CEP55, Angiogenesis, H3K4me3

## Abstract

**Background:**

This study aims to clarify the mechanistic action of long non-coding RNA (lncRNA) SNHG12 in the development of renal cell carcinoma (RCC), which may be associated with promoter methylation modification by KMT2B and the regulation of the E2F1/CEP55 axis.

**Methods:**

TCGA and GEO databases were used to predict the involvement of SNHG12 in RCC. Knockdown of SNHG12/E2F1/CEP55 was performed. Next, SNHG12 expression and other mRNAs were quantified by RT-qPCR. Subsequently, CCK-8 was used to detect cell proliferation. Wound healing assay and Transwell assay were used to detect cell migration and invasion, respectively. The in vitro angiogenesis of human umbilical vein endothelial cells (HUVECs) was explored by matrigel-based capillary-like tube formation assay. ChIP assay was used to detect H3K4me3 in SNHG12 promoter region. The binding of E2F1 to CEP55 promoter region was analyzed with ChIP and dual luciferase reporter assays. RIP assay was used to detect the binding of SNHG12 to E2F1. Finally, the effect of SNHG12 on the tumor formation and angiogenesis of RCC was assessed in nude mouse xenograft model.

**Results:**

SNHG12 was highly expressed in RCC tissues and cells, and it was related to the poor prognosis of RCC patients. SNHG12 knockdown significantly inhibited RCC cell proliferation, migration, and invasion and HUVEC angiogenesis. KMT2B up-regulated SNHG12 expression through modifying H3K4me3 in its promoter region. In addition, SNHG12 promoted CEP55 expression by recruiting the transcription factor E2F1. Knockdown of SNHG12 blocked E2F1 recruitment and down-regulated the expression of CEP55, thereby inhibiting tumor formation and angiogenesis in nude mice.

**Conclusion:**

The evidence provided by our study highlighted the involvement of KMT2B in up-regulation of lncRNA as well as the transcription of CEP55, resulting in the promotion of angiogenesis and growth of RCC.

**Supplementary Information:**

The online version contains supplementary material available at 10.1186/s12935-022-02607-w.

## Background

Renal cell carcinoma (RCC) is the main pathological type of kidney cancer, accounting for 70% to 90% [1]. Epidemiological survey shows that its morbidity and mortality have both been on the rise worldwide in recent years [2]. RCC is a highly concealed malignant tumor originating from the renal tubular epithelium. Only 10% of patients present with the “classic triad” (flank pain, gross hematuria, and a palpable renal mass) [3]. Due to the lack of biomarkers for early diagnosis and prognosis of RCC, most RCC patients are diagnosed at the middle and late stages, accompanied by local spread and distant metastasis [4]. The main treatment of RCC is radical or partial nephrectomy followed by chemotherapy and/or radiotherapy. In addition, 20%—40% of patients have recurrence and/or distant metastasis after surgery. Although progresses have been made in the diagnosis and treatment of RCC in the past decades, RCC is still one of the most drug-resistant malignancies and a common cause of cancer-related deaths [5]. Therefore, it is urgent to explore the molecular mechanism of RCC occurrence and development, to identify new and reliable biomarkers of RCC and to develop new therapeutic targets for early diagnosis and treatment of RCC.

Long non-coding RNA (lncRNA) is a type of non-coding RNA with a length greater than 200 nucleotides. It is involved in the multi-level regulation of gene expression and its abnormal expression and mutation are usually closely related to tumorigenesis and metastasis [6–8]. In addition, lncRNA can be specifically expressed in cancer and stably exist in body fluids [9–11], which can be used as a new type of cancer biomarkers and therapeutic targets. Some lncRNAs can encode small nucleolar RNA and are called small nucleolar RNA host genes (SNHGs). Of them, SNHG12 has been reported to be up-regulated in human endometrial cancer [12], bladder cancer [13], nasopharyngeal cancer [14], colorectal cancer [15], lung adenocarcinoma [16], breast cancer [17], liver cancer [18], and clear cell RCC [19], and plays an important role in the proliferation and migration of cancer cells. SNHG12 expression is upregulated in RCC tumor tissues while its knockdown markedly inhibits RCC cell viability and invasion, while increasing apoptosis [20]. In addition, SNHG12 promotes the progression of RCC and RCC sunitinib resistance [21]. Meanwhile, SNHG12 can promote the malignancy of clear cell RCC by competitively binding with miR-30a-3p and consequently releasing the expression of its downstream cancer-related genes [19]. The methylation of the lncRNA promoter can regulate the expression of lncRNAs, which is related to the occurrence of many diseases. For example, in clear cell RCC, the methylation status of two CpG sites is negatively correlated with the expression of SNHG3 and SNHG15, suggesting that DNA hypomethylation may play an important role in promoting the transcription of SNHG3 and SNHG15 [22]. SNHG11 binds to the HRE site in the gene promoter, and promotes gene transcription and tumor invasion and metastasis of colorectal cancer through the SNHG11/HIF-1α pathway [23]. CpG methylation in the promoter region of SNHG12 promotes the competitive binding of SNHG12 with miR-129-5p, regulates the MAPK/ERK pathway and G1/S cell cycle transition, thereby affecting the resistance of glioblastoma cells to temozolomide [24]. However, the role of SNHG12 regulated by DNA methylation in RCC is still unclear.

In this study, we explored the role of SNHG12 promoter methylation in RCC development through series loss- and gain-of-function experiments. Our findings may provide evidence for identifying new molecular targets for the diagnosis and treatment of RCC.

## Materials and methods

### Bioinformatics analysis

RNA sequencing data and corresponding clinical data of RCC tissue and normal tissue samples were downloaded from TCGA database through UCSC Xena (https://xena.ucsc.edu/). The GSE71963 dataset was obtained from GEO database (https://www.ncbi.nlm.nih.gov/gds). In total, we obtained 535 RCC tissue samples and 72 normal tissue samples from TCGA, as well as 32 RCC samples and 16 normal tissues samples from GSE71963 dataset. The R language “limma” package (http://www.bioconductor.org/packages/ release/bioc/html/limma.html) was used to screen differentially expressed lncRNAs, with |logFC|> 1 and *p* < 0.05. Kaplan–Meier survival analysis was performed using the R software “survival” package (http://bioconductor.org/packages/survival/), with *p* < 0.05. ENCORI database (http://starbase.sysu.edu.cn/index.php) was used for correlation analysis. LncMAP database (http://bio-bigdata.hrbmu.edu.cn/LncMAP/) was used to predict the transcription factors of SNHG12 regulating centrosome protein 55 (CEP55) in RCC. The UCSC database (http://genome-asia.ucsc.edu/) was used to analyze the epigenetic modification of the SNHG12 promoter region.

### Study cohort

RCC tissues and the corresponding precancerous tissue samples were collected from RCC patients (n = 46; 30 males and 16 females; aged 39–71 years with a mean age of 56.11 ± 10.29 years) at Mianyang Central Hospital from January 2017 to January 2019. All patients were confirmed to have RCC by surgery and pathological analysis. The inclusion criteria: 1) Patients with pathologically confirmed RCC cases; 2) Patient did not receive any chemotherapy, radiotherapy, or other antitumor treatment before operation; 3) Patients had complete clinical data. The exclusion criteria: 1) Patients without pathological confirmation; 2) Patients with recurrence and distant metastasis after treatment; 3) Patients with a history of mental illness; 4) Patients with dysfunction of the heart, liver, pancreas, and other important organs; 5) Patients with respiratory and circulatory diseases; 6) Patients with non-RCC tumors. Tissue samples were frozen in liquid nitrogen and stored at − 80 °C. Among the included patients, 33 cases at stage I-II, 13 cases at stage III-IV, 28 cases at stage G1-2, and 18 cases at stage G3; 11 cases with lymph node metastasis and 35 without lymph node metastasis. The current study was approved by the Ethics Review Committee of Mianyang Central Hospital (P2020030) and performed in strict accordance with the *Declaration of Helsinki*. All participants signed informed consent documentation.

### Cell culture and transfection

Human umbilical vein endothelial cells (HUVECs), purchased from Zhong Qiao Xin Zhou Biotechnology (DFSC-EC-01; Shanghai, China), were cultured in 500 mL endothelial cell basal medium containing 25 mL fetal bovine serum, 5 mL endothelial cell growth factor and 5 mL penicillin/streptomycin solution. RCC cell lines A498, 786-O, Caki-1, and 769-P, and human normal kidney cell line HK-2 purchased from ATCC (MA, VA) were cultured in modified Eagle’s medium (Gibco Company, Grand Island, NY) containing 10.0% FBS (Gibco) and 1.0% antibiotics (100 U/mL penicillin and 100 mg/mL streptomycin).

Cells were transfected with plasmids of sh-SNHG12 (short hairpin RNA-SNHG12), oe-SNHG12 (SNHG12 overexpression), sh-KMT2B, oe-E2F1, oe-SNHG12 + sh-E2F1, sh-E2F1 + oe-CEP55, using the Lipofectamine 2000 reagent (Invitrogen, Carlsbad, CA). Corresponding negative control (NC) groups were set up. The plasmids were all constructed by GenePharma Co., Ltd. (Shanghai, China), and the plasmid concentration was 50 ng/mL. The shRNA sequences are shown in Additional file 1: Table S1.

### CCK-8

After 48 h of transfection, cells were seeded into 96-well plates at a density of 1.0 × 10^5^ cells/mL (100 μL/well). After routine culture overnight, the cells were treated according to CCK-8 kit (Beyotime, Shanghai, China), and the cell viability was detected at 24 h, 48 h, and 72 h. The OD450 was detected with a microplate reader.

### Wound healing assay

Cells were seeded in a 6-well plate at 5 × 10^5^ cells/mL and cultured for 24 h. Next, a 200 μL sterile pipette tip was used to make a scratch on the cells perpendicular to the horizontal line on the back. Cells continued to culture in the renewed serum-free medium. At 0 and 48 h, the scratch distance was measured and recorded under an optical microscope (DM500; Leica Wetzlar, Germany), and images were acquired under an inverted microscope. Image J software was used to determine the width of the scratches in each well. The relative distance of cell migration to scratch area was measured, and the actual migration distance was calculated according to the scratch area distance of cells.

### Transwell assay

The Transwell upper chamber (Yanhui Biotechnology, Shanghai, China) was pre-coated with ECM gel (Sigma-Aldrich). After starving culture for 24 h, the cells were added to the upper chamber at 2.5 × 10^5^ cells/mL (0.2 mL in total). In the lower chamber, 700 μL of pre-cooled DMEM medium containing 10% FBS was added. The chamber was then incubated in a 37 °C, 5% CO_2_ saturated humidity incubator. After 24 h, the cells in the lower chamber were fixed with methanol, and stained with 0.1% crystal violet. Invaded cells were photographed and counted using randomly selected 5 visual fields in each chamber under an inverted microscope. Cell migration experiments were conducted using the same procedures except for ECM gel.

### Matrigel-based capillary-like tube formation in vitro

RCC cells were transfected as above described. After 48 h, the cell supernatant was collected. The tumor-conditioned medium was prepared according to the ratio of 4:5:1 (tumor supernatant: DMEM medium: FBS). HUVECs were seeded into a 96-well plate pre-coated with Matrigel and incubated with tumor-conditioned medium for 8 h. Finally, 4 fields of view were randomly selected from each well and the tube length was quantified under a phase-contrast microscope.

### Fractionation of nuclear/cytoplasmic RNA

Nuclear and cytoplasmic fractions of cells were isolated using the PARIS kit (Life Technologies, Carlsbad, CA). RCC cells were trypsinized and centrifuged at 500 g and 4 °C for 5 min. The pellet was rinsed with PBS, added with 500 μL Cell Fractionation Buffer, gently dispersed, left to stand on ice for 5–10 min, and centrifuged at 500 g and 4 °C for 5 min. Next, the supernatant (cytoplasm) was transferred into a new 2 mL sterile enzyme-free tube, and centrifuged at 500 g and 4 °C for 5 min. The pellet (nucleus) was mixed with 500 μL Cell Fractionation Buffer, 500 μL of 2 × Lysis/binding solution at room temperature, pre-cooled 500 μL Cell Disruption Buffer, and 500 μL of anhydrous ethanol. The adsorption column was put into a collection tube, which was added with 700 μL of reaction solution each time, and centrifuged at 12,000 *g* for 30 s. The sample was subsequently eluted with 40 μL of Elution solution by centrifugation at 12,000 *g* for 30 s and with 10 μL of Elution solution. RT-qPCR was used to detect the expression of SNHG12, with GAPDH as the cytoplasmic marker and U6 as the nuclear marker.

### Chromatin immunoprecipitation (ChIP)

786-O cells transfected with sh-NC or sh-KMT2B were fixed with 1.0% formaldehyde and incubated for 10 min to generate DNA–protein crosslinks, which were then halted by glycine. Next, the cells were subjected to ultrasonic treatment to obtain the DNA fragments. For ChIP, the supernatant was incubated with the rabbit anti-IgG antibody (ab109489, 1:100, serving as NC), anti-H3K4me3 antibody (1: 1000, ab8580), and anti-E2F1 antibody (1: 500, ab179445) (Abcam, Cambridge, UK) at 4 °C overnight. The endogenous DNA–protein complexes were precipitated with Protein Agarose/Sepharose (Sangon biotech, Shanghai, China). The DNA fragments were extracted with phenol/chloroform. RT-qPCR was used to detect the enrichment of H3K4me3 in the SNHG12 promoter and E2F1 in the CEP55 promoter.

### RNA binding protein immunoprecipitation (RIP)

RIP kit (Millipore) was used to detect the binding of SNHG12 and E2F1 protein. The 786-O cells were lysed with RIPA buffer (P0013B, Beyotime) containing protease inhibitor for 5 min. A portion of cell lysates were taken out as Input, and another was incubated with antibodies against E2F1 (1:50, ab179445, Abcam) and NC IgG (ab172730, 1:100, Abcam) for immunoprecipitation. The antibody-magnetic bead complexes, which were prepared by incubating magnetic beads with the above antibodies, were incubated with the supernatant at 4 °C overnight. After that, the samples were digested with proteinase K and RNA was extracted for subsequent RT-qPCR detection.

### Dual luciferase reporter assay

The potential binding site of E2F1 in the promoter region of CEP55 was analyzed through the JASPAR website (http://jaspar.genereg.net). After that, the pGL3-CEP55-WT and pGL3-CEP55-MUT plasmids were constructed with wild type (WT) and mutant type (MUT) binding sequences of E2F1, respectively. Then, these plasmids were co-transfected with oe-NC and oe-E2F1 into the HEK-293 T cells (ATCC). After 48 h, the Dual-Luciferase Reporter Assay System kit (Promega) and the TD-20/20 Luminometer were used to detect the luciferase activity.

### RT-qPCR

The nucleus and cytoplasm of RCC cells were separated using PARIS kit (Life Technologies, Carlsbad, CA). The total RNA of cells and tissues as well as nucleus and cytoplasm of RCC cells was extracted by Trizol (Invitrogen). RT-qPCR was conducted as reported previously [25]. The primer sequences are shown in Additional file 1: Table S2.

### Western blot

Total protein of tissues or cells was extracted with RIPA (P0013B, Beyotime). Protein concentration was determined using BCA kit (ThermoFisher Scientific). After separation by polyacrylamide gel electrophoresis, 50 μg protein was transferred to PVDF membranes and blocked with 5% BSA for 1 h. The detailed experimental method was similar to a previous report [26].

### Nude mouse xenograft model

BALB/c female nude mice (n = 30; 3–4 weeks old; weighing: 14–18 g; J004, Nanjing Junke Bioengineering Co., Ltd., Jiangsu, China) were housed in the SPF laboratory at 18–22 °C and 50–60% humidity under a 12-h light/dark cycle. The health of the mice was observed before the experiment. The mice were randomly grouped and inoculated with 786-O cell suspension containing 2 × 10^7^ cells/mL (0.1 mL) stably transfected with sh-SNHG12 + oe-E2F1, sh-E2F1 + oe-CEP55, and corresponding NCs (n = 6 for mice upon each treatment). The tumor volume was measured weekly. The tumor volume was calculated by length × width^2^ × 0.5. Five weeks after inoculation, mice were euthanized by intraperitoneal injection of overdose pentobarbital sodium (100 mg/kg). The tumors were dissected, and weighed, with the tumor length and width measured. The tumor tissue was frozen in liquid nitrogen and stored at − 80 °C for subsequent experiments. Animal experiments were approved by the Animal Ethics Committee of Mianyang Central Hospital (P2020030). All efforts were made to minimize animal suffering.

### Immunohistochemistry

Paraffin sections of tumor tissues were dewaxed, hydrated with gradient alcohol and antigen retrieved in water bath. Next, the sections were blocked with normal goat serum blocking solution (C-0005, Shanghai Haoran Biotechnology Co., Ltd., Shanghai, China) and incubated with anti-VEGF antibody (ab72807, 1:50) at 4 °C overnight. After that, sections were incubated with goat anti-rabbit IgG secondary antibody (ab150077, 1:100), developed with DAB, and counterstained with hematoxylin. The results were evaluated by two experienced pathologists in a blinded manner. The positive rate of VEGF was calculated as the ratio of positive cells to total number of cells. Specific experiment procedures were similar to previous reports [26, 27].

### Statistical analysis

All data were analyzed using SPSS 21.0 software, and expressed as Mean ± SD. Statistical analysis for data included paired t-test for paired data comparison between two groups, unpaired t-test for unpaired data comparison between two groups, one-way ANOVA with Tukey’s test for data comparison between multiple groups, two-way ANOVA for comparison of OD values at different time points or Bonferroni-corrected repeated measures ANOVA for comparison of tumor data at different time points. *p* < 0.05 indicates that the difference was statistically significant.

## Results

### SNHG12 is up-regulated in RCC tissues and cell lines

**Fig. 1:**
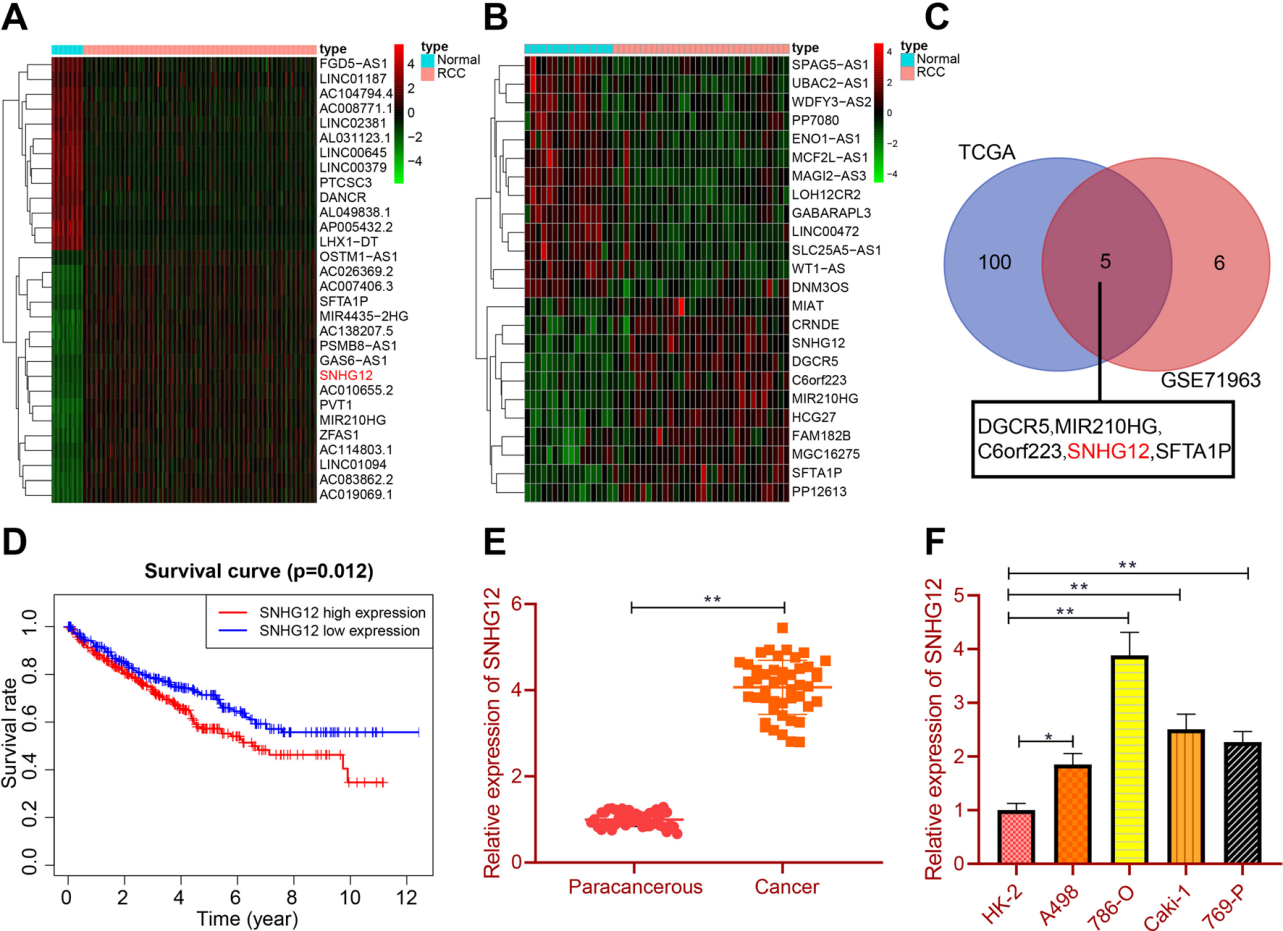
High expression of SNHG12 in RCC tissues and cells. **A** A heat map of the first 30 significantly differentially expressed lncRNAs between 535 RCC samples and 72 normal tissue samples from TCGA database. **B** A heat map of significantly differentially expressed lncRNAs between 32 RCC samples and 16 normal tissue samples in the GSE71963 dataset. **C** Venn diagram of the up-regulated lncRNAs in TCGA database and the GSE71963 dataset. **D** Kaplan–Meier overall survival curve of RCC patients with high and low SNHG12 expression in TCGA database. **E** RT-qPCR analysis of SNHG12 expression in RCC tissues and normal adjacent tissues (n = 46). **F** RT-qPCR analysis of SNHG12 expression in HK-2 cells and RCC cell lines. **p < 0.01. All cell experiments were repeated three times

Following intersection analysis of the differentially expressed lncRNAs in the RCC tissue samples in TCGA database and the GSE71963 dataset, 5 highly expressed lncRNAs were identified in RCC tissue samples (Fig. 1A–C). However, further survival analysis showed that only SNHG12 was associated with the prognosis of RCC whereby RCC patients with high SNHG12 expression had poorer prognosis compared with those with low SNHG12 expression (Fig. 1D).

Next, compared with paracancerous tissues, SNHG12 was upregulated in RCC tissues (Fig. 1E). In addition, SNHG12 had no significant relation with patient sex and age, but had significant association with tumor grade, TNM stage, and lymph node metastasis (Additional file 1: Table S3).

Compared with HK-2 cells, RCC cell lines (A498, 786-O, Caki-1, and 769-P) showed up-regulated SNHG12 expression, with the 786-O cells showing the highest SNHG12 expression (Fig. 1F) and thus selected for further assays.

Collectively, the above findings suggested the abundant SNHG12 expression in RCC tissues and cell lines.

### SNHG12 knockdown inhibits proliferation, migration, and invasion of RCC cells and HUVEC angiogenesis

**Fig. 2:**
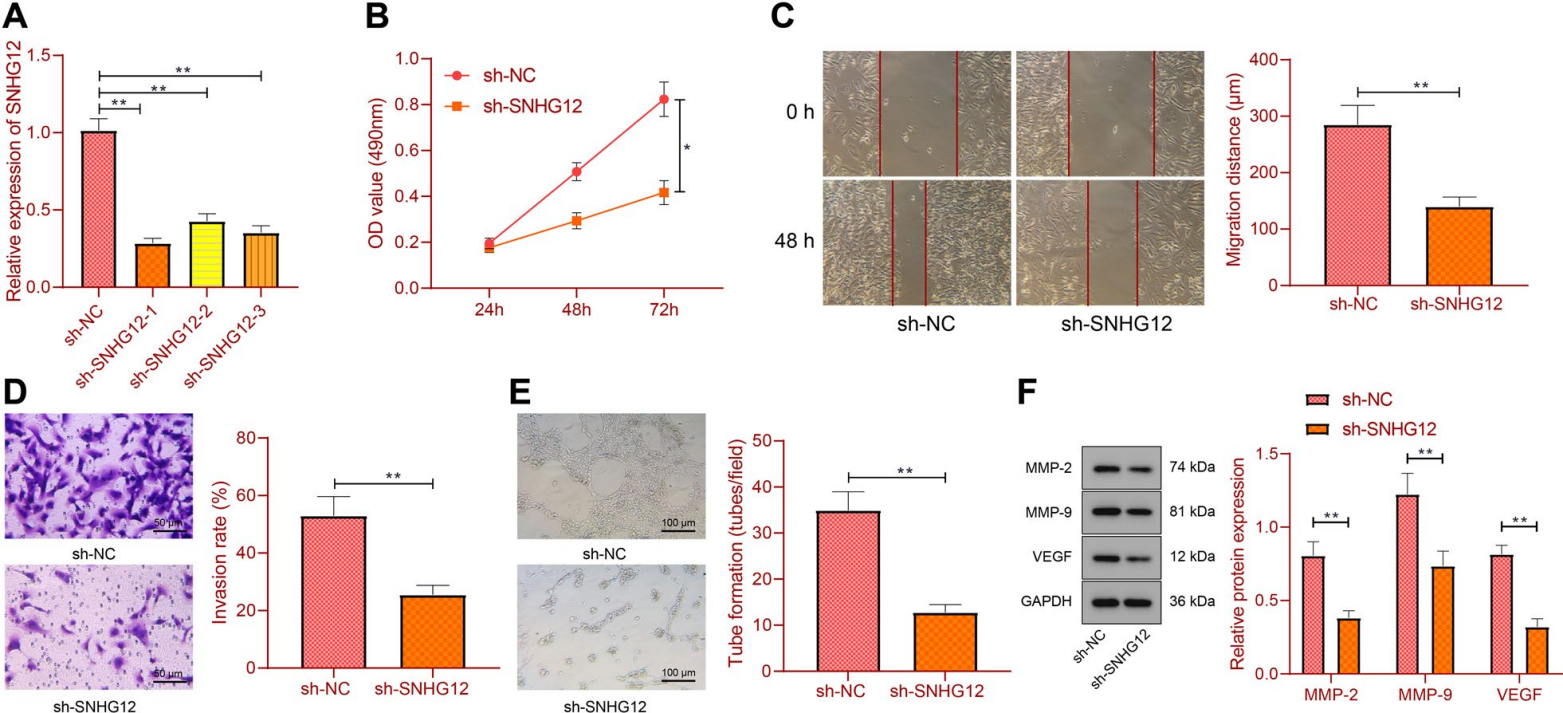
The effect of knockdown of SNHG12 on RCC cells. **A** The knockdown efficiency of SNHG12 in 786-O cells was tested by RT-qPCR. **B** The proliferation ability of 786-O cells was detected by CCK-8 assay. **C** 786-O cell migration ability was detected by wound healing assay. **D** 786-O cell invasion ability was analyzed by Transwell assay. **E** Detection of capillary-like tube formation of HUVECs in vitro. **F** Detection of MMP-2, MMP-9, and VEGF protein expression in 786-O cells by Western blot. *p < 0.05, **p < 0.01. All cell experiments were repeated three times

SNHG12 was knocked down by three shRNA sequences in 786-O cells, RT-qPCR results showed that the three shRNA sequences successfully reduced the expression of SNHG12, with sh-SNHG12-1 presenting the highest knockdown efficiency (Fig. 2A) and thus used for subsequent experiments.

Moreover, CCK-8, wound healing assay and Transwell assay results showed that SNHG12 knockdown inhibited RCC cell proliferation, migration and invasion (Fig. 2B–D). In addition, in vitro capillary-like tube formation experiments showed that knockdown of SNHG12 inhibited capillary-like tube formation, thus suppressing HUVEC angiogenesis (Fig. 2E). Western blot data further indicated that knockdown of SNHG12 decreased the protein expression of MMP-2, MMP-9 and VEGF in 786-O cells (Fig. 2F).

Thus, SNHG12 knockdown can repress the proliferation, migration, and invasion of RCC cells and HUVEC angiogenesis.

### KMT2B up-regulates SNHG12 expression by regulating the H3K4me3 modification of the SNHG12 promoter

**Fig. 3:**
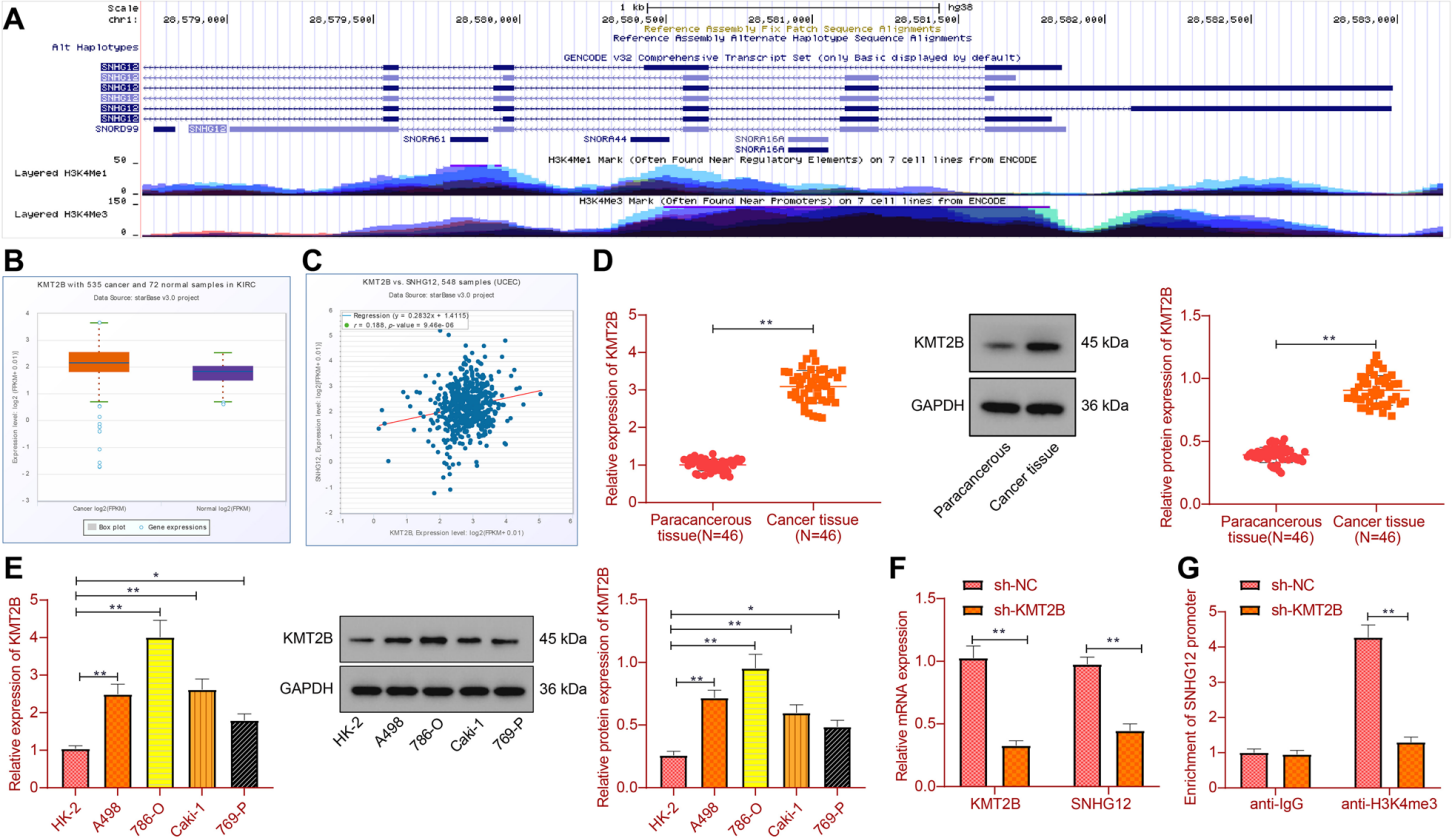
The effect of KMT2B on H3K4me3 modification in the promoter region of SNHG12. **A** The enrichment of H3K4me3 modification in the promoter region of SNHG12 was analyzed by the UCSC database. **B** The expression of KMT2B in RCC was analyzed by ENCORI database (RCC = 535, Normal = 72). **C** Through the ENCORI database, the correlation between KMT2B and SNHG12 in RCC was analyzed. **D** The expression of KMT2B in RCC tissues and normal adjacent tissues was detected by RT-qPCR and Western blot (n = 46). **E** The expression of KMT2B in RCC cells was detected by RT-qPCR and Western blot. **F** The expression of KMT2B and SNHG12 in 786-O cells transfected with sh-KMT2B detected by RT-qPCR. **G** ChIP was used to detect the enrichment of H3K4me3 in the promoter region of SNHG12 in 786-O cells transfected with sh-KMT2B. *p < 0.05, **p < 0.01. All cell experiments were repeated three times

As shown in Fig. 3A, there was a large amount of H3K4me3 enrichment in the SNHG12 promoter. It is reported that KMT2B mediates the transcriptional activation of H3K4me3 modification [28]. Moreover, through ENCORI database analysis, KMT2B was highly expressed in RCC (Fig. 3B) and positively correlated with SNHG12 expression (Fig. 3C). Therefore, KMT2B may regulate SNHG12 by mediating the H3K4me3 modification of its promoter region.

KMT2B mRNA and protein expression was increased in RCC tissues compared with normal adjacent tissues (Fig. 3D). Compared with human normal kidney cell line HK-2, KMT2B and protein expression in RCC cells was increased (Fig. 3E). Next, we knocked down KMT2B in 786-O cells and found reduced KMT2B and SNHG12 expression (Fig. 3F). ChIP showed that knockdown of KMT2B reduced modification of SNHG12 promoter H3K4me3 (Fig. 3G).

The aforementioned data supported that KMT2B could increase SNHG12 expression through the H3K4me3 modification of the SNHG12 promoter.

### SNHG12 recruits E2F1 to promote CEP55 expression in RCC cells

**Fig. 4:**
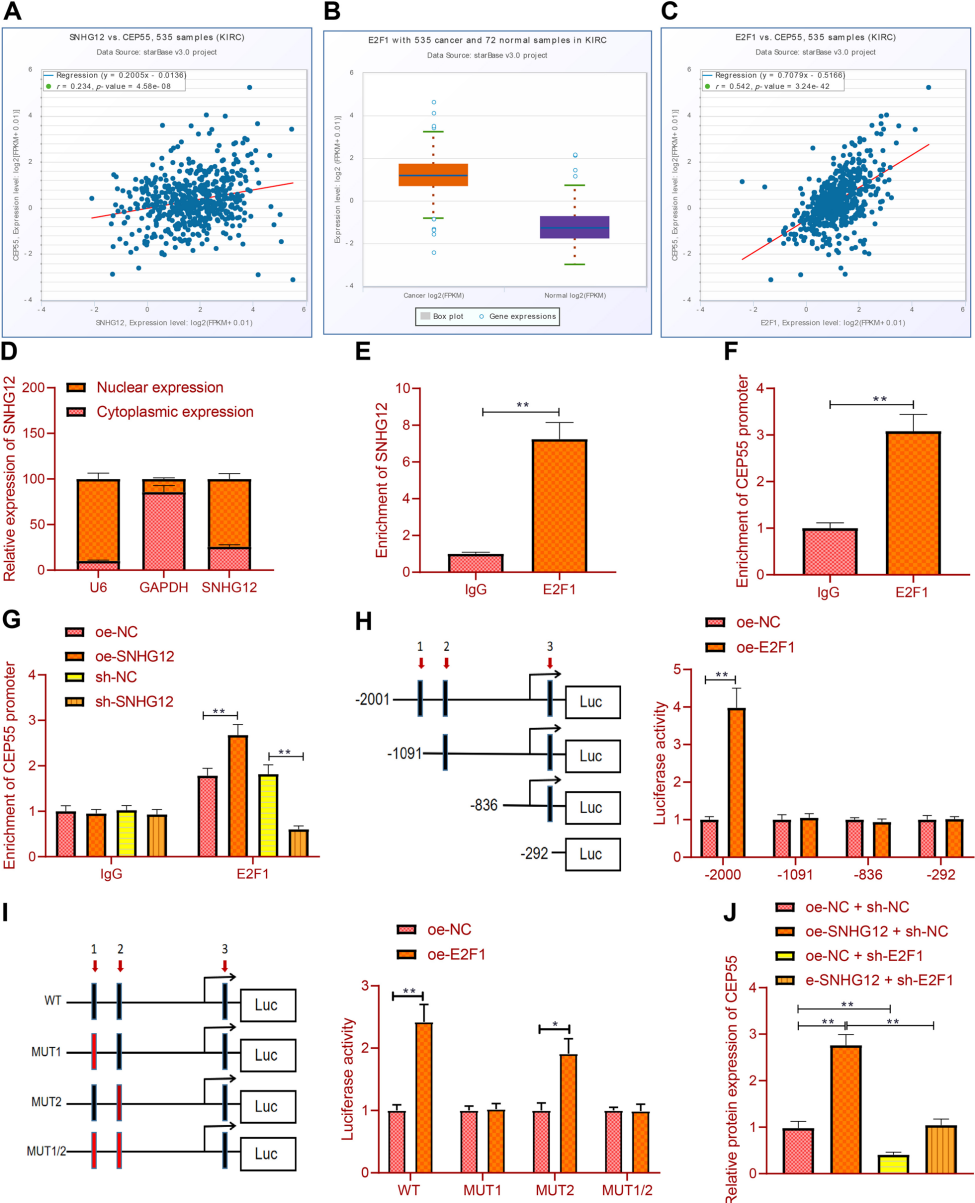
SNHG12 recruits transcription factor E2F1 to elevate CEP55 transcription. **A** The correlation between SNHG12 expression and CEP55 expression in RCC samples was analyzed through ENCORI database. **B** The expression of E2F1 in RCC samples was analyzed through the ENCORI database (RCC = 535, Normal = 72). **C** The correlation between E2F1 expression and CEP55 expression in RCC samples was analyzed through the ENCORI database. **D** Cellular localization of SNHG12 was analyzed by fractionation of nuclear/cytoplasmic RNA. **E** The binding of SNHG12 and E2F1 was detected by RIP. **F** Detection of E2F1 binding to the CEP55 promoter region by ChIP. **G** After overexpression of SNHG12, the binding of E2F1 to the CEP55 promoter region was detected by ChIP. **H** The binding of transcription factor E2F1 to the promoter region of the target gene CEP55 was detected by dual luciferase reporter assay. I: Three sites in the promoter region of CEP55. **J** The expression of CEP55 in 786-O cells transfected with oe-SNHG12, sh-E2F1 or both was detected by RT-qPCR. *p < 0.05, **p < 0.01. All cell experiments were repeated three times

In RCC, SNHG12 expression was positively correlated with CEP55 expression in RCC samples (Fig. 4A). Analysis with LncMAP database showed that E2F1 was one of the transcription factors involved in the regulation of SNHG12 on CEP55 in RCC (Additional file 1: Table S4). Additionally, ENCORI database analysis showed that E2F1 was highly expressed in RCC samples (Fig. 4B) and positively correlated with CEP55 expression (Fig. 4C). Therefore, SNHG12 may regulate CEP55 through E2F1.

To further verify this hypothesis, we showed that SNHG12 was mainly located in the nucleus (Fig. 4D). RIP results showed enriched SNHG12 when using anti-E2F1 antibody (Fig. 4E). Meanwhile, E2F1 was highly enriched in the CEP55 promoter region (Fig. 4F). Furthermore, overexpression of SNHG12 promoted the enrichment of E2F1 in the CEP55 promoter region (Fig. 4G). Then, we predicted the binding site between E2F1 and CEP55 promoter region through Jaspar (Additional file 1: Table S5). Dual luciferase reporter assay showed that the luciferase activity was enhanced after overexpression of E2F1 when the full-length promoter was retained, while the luciferase activity did not change after truncation of site 1. In order to verify that site 1 may be the main site for E2F1 to act on CEP55, we further mutated site 1 and site 2, and found that when site 1 or sites 1 and 2 were mutated at the same time, there was no alteration in the luciferase activity in the presence of overexpression of E2F1, while the luciferase activity was increased in the mutation site 2 (Fig. 4H–I). It was revealed that site 1 was the main site for E2F1 to promote the transcriptional activity of CEP55.

Finally, in the 786-O cells, CEP55 expression was increased by SNHG12 overexpressing, but reduced by further silencing E2F1. When overexpressing SNHG12 and silencing E2F1 at the same time, CEP55 expression was reduced (Fig. 4J).

The above results indicated that the SNHG12 elevated CEP55 expression in RCC cells by recruiting E2F1 in RCC cells.

### SNHG12 recruits E2F1 to promote RCC cell proliferation, migration, and invasion and HUVEC angiogenesis

**Fig. 5:**
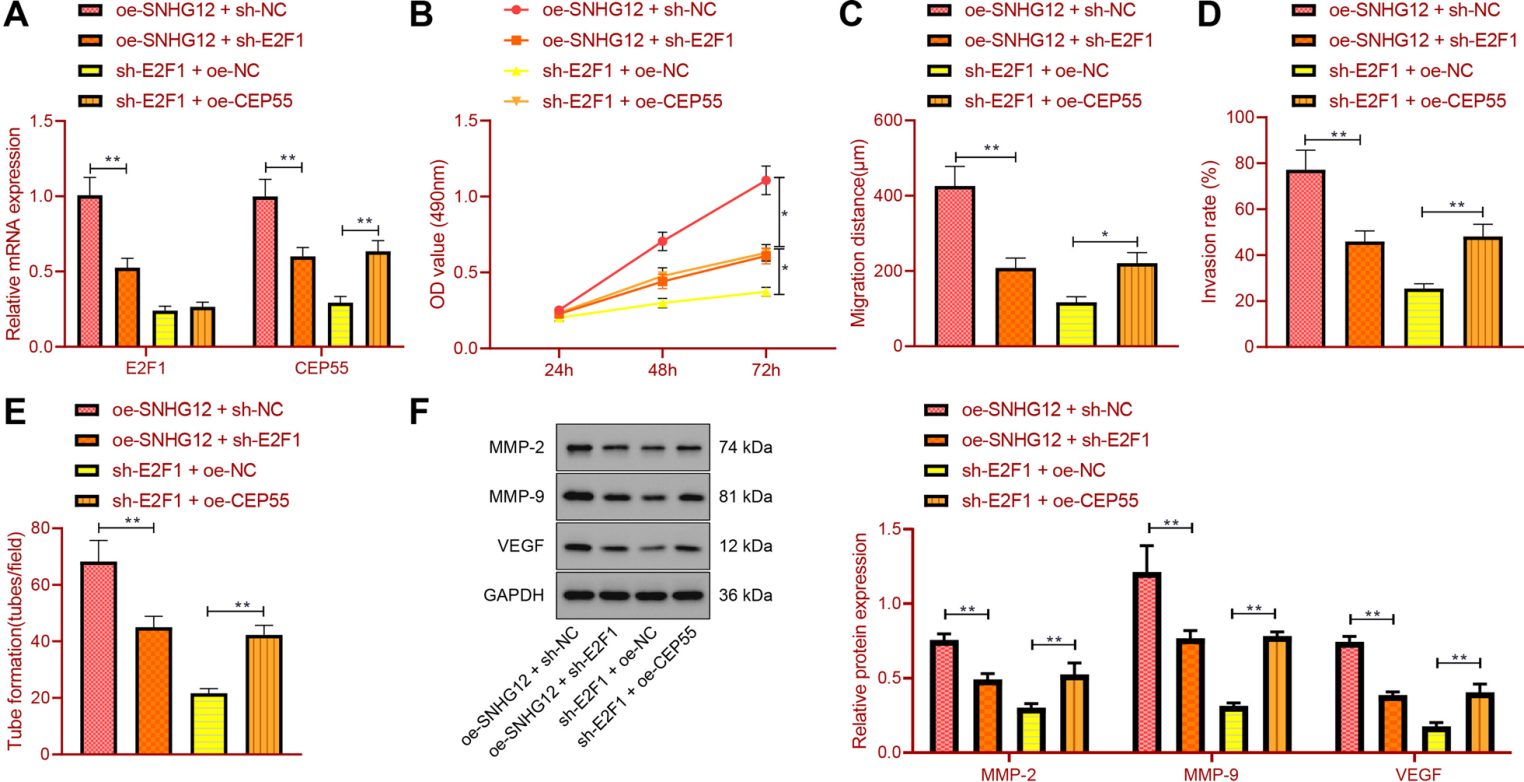
The effect of SNHG12/E2F1/CEP55 on RCC cell proliferation, migration, invasion and HUVEC angiogenesis. 786-O cells were transfected with oe-SNHG12 + sh-NC, oe-SNHG12 + sh-E2F1, sh-E2F1 + oe-NC or sh-E2F1 + oe-CEP55. **A** The expression of E2F1 and CEP55 in 786-O cells was detected by RT-qPCR. **B** The proliferation ability of 786-O cells was detected by CCK-8. **C** Detection of 786-O cell migration by wound healing assay. **D** Detection of 786-O cell invasion by Transwell assay. **E** Analysis of the angiogenesis of HUVECs by in vitro by capillary-like tube formation assay. **F** Detection of the expression of MMP-2, MMP-9 and VEGF in 786-O cells by Western blot. **p < 0.01. All cell experiments were repeated three times

Compared with oe-SNHG12 + sh-NC, E2F1 and CEP55 expression was decreased by oe-SNHG12 + sh-E2F1. Compared with sh-E2F1 + oe-NC, E2F1 expression remained not changed by sh-E2F1 + oe-CEP55 treatment, but CEP55 expression was elevated (Fig. 5A).

In addition, compared with SNHG12 overexpression alone, further E2F1 silencing reduced cell proliferation, migration and invasion and capillary-like tube formation ability while CEP55 overexpression reversed the above effects of E2F1 silencing (Fig. 5B–E). Meanwhile, the protein expression of MMP-2, MMP-9, and VEGF was decreased in the presence of oe-SNHG12 + sh-E2F1 compared with oe-SNHG12 alone, while the trend was opposite in the presence of sh-E2F1 + oe-CEP55 compared with the individual sh-E2F1 (Fig. 5F).

Taken together, these results demonstrated that SNHG12 favored RCC cell malignant phenotypes and HUVEC angiogenesis by recruiting E2F1.

### Knockdown of SNHG12 inhibits RCC growth and angiogenesis in vivo

**Fig. 6:**
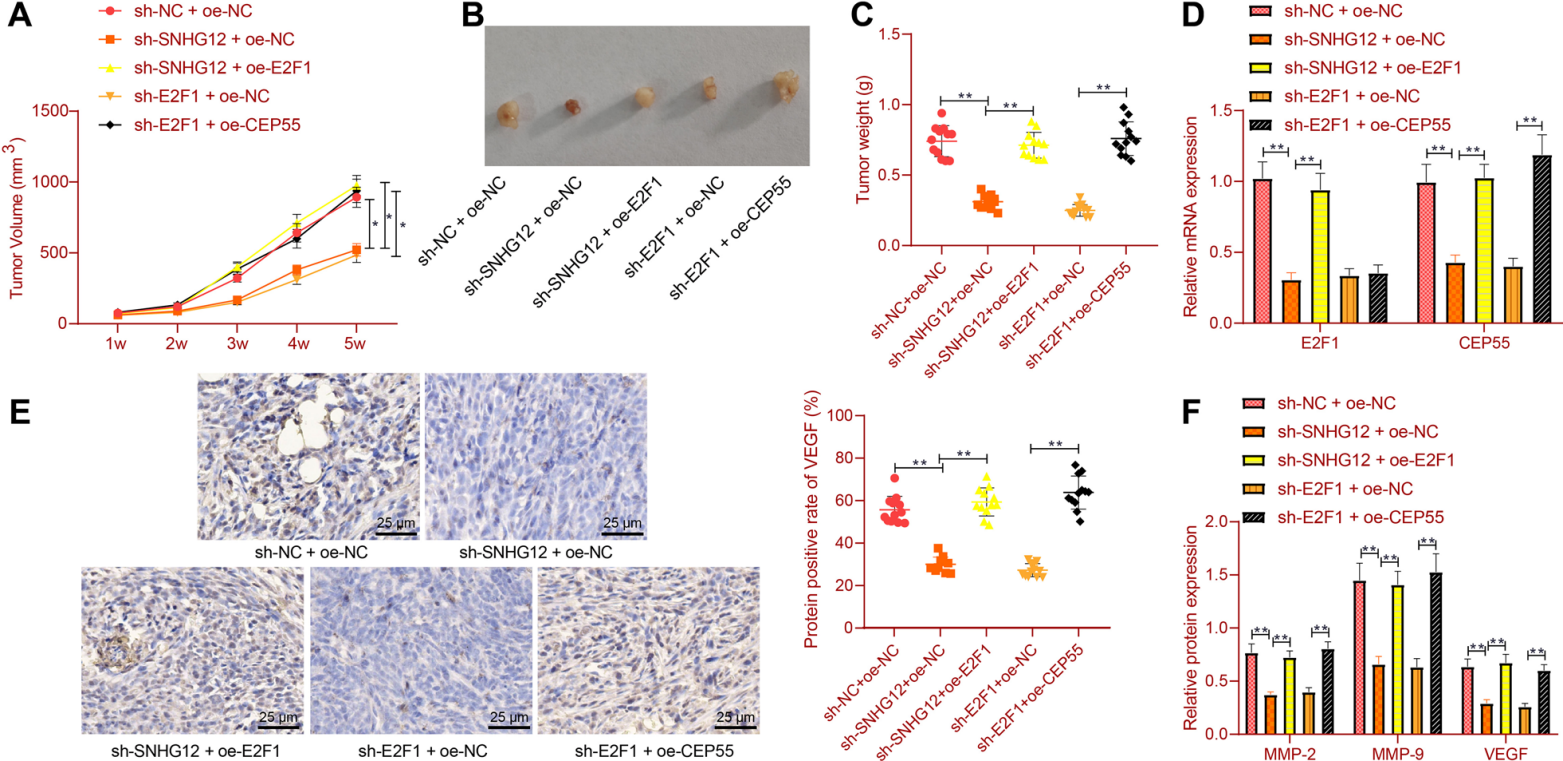
The effect of SNHG12/E2F1/CEP55 on RCC growth and angiogenesis in vivo. Nude mice were treated with sh-SNHG12 + oe-NC, sh-SNHG12 + oe-E2F1, sh-E2F1 + oe-NC or sh-E2F1 + oe-CEP55. **A** Tumor volume of mice. **B** Representative images showing xenografts in mice. **C** Tumor weight of mice. **D** The mRNA expression of *E2F1* and *CEP55* in tumor tissues of mice was detected by RT-qPCR. **E** The protein expression of VEGF in tumor tissues of mice was detected by immunohistochemistry. **F** Detection of the protein expression of MMP-2, MMP-9 and VEGF in tumor tissues of mice by Western blot; *p < 0.05, **p < 0.01. n = 6

As shown in Fig. 6A–C, compared with the NC, SNHG12 silencing slowed down tumor growth and reduced tumor weight, while further E2F1 overexpression boosted tumor growth and increased tumor weight. Compared with silencing E2F1 alone, simultaneous silencing E2F1 and overexpressing CEP55 enhanced tumor growth.

Compared with NC, silencing SNHG12 reduced E2F1 and *CEP55* expression, while further E2F1 overexpression reversed this trend. Compared with silencing E2F1 alone, simultaneous silencing E2F1 and overexpressing CEP55 up-regulated *CEP55* (Fig. 6D).

Immunohistochemical detection showed that compared with NC, silencing SNHG12 decreased VEGF protein expression, which was reversed by further E2F1 overexpression. Compared with silencing E2F1 alone, simultaneous silencing E2F1 and overexpressing CEP55 increased VEGF expression (Fig. 6E). Meanwhile, compared with NC, silencing SNHG12 reduced MMP-2, MMP-9, and VEGF protein expression, which was neutralized by further E2F1 up-regulation. Compared with silencing E2F1 alone, their expression was promoted after simultaneous silencing E2F1 and overexpressing CEP55 (Fig. 6F).

Overall, these data revealed that silencing of SNHG12 blocked its binding to E2F1 and downregulated CEP55 expression, ultimately inhibiting RCC growth and angiogenesis.

## Discussion

RCC is a heterogeneous tumor that originates from the renal parenchyma and is one of the deadliest malignant tumors in the urinary system [29]. So far, the mechanism underlying the occurrence and development of RCC is still not fully understood. RCC-related biomarkers are less studied, and RCC early diagnosis is difficult. In addition, RCC responds poorly to conventional chemotherapy and radiotherapy, and there is a lack of targeted therapy drugs for RCC [30]. However, if diagnosed early, patients with local RCC can be treated by nephrectomy (partial or total nephrectomy). The treatment effect of TI and T2 stage surgery is better, which can not only improve the quality of life, but also the 5- to 10-year survival rate of RCC patients [31]. Therefore, further understanding of the pathogenesis of RCC may help the diagnosis and treatment of RCC patients. Here, in this study, we explored the regulation of SNHG12 by KMT2B and the downstream factors of SNHG12 (including E2F1 and CEP55) involved in its effect on RCC.

SNHG12 exerts a carcinogenic effect in a variety of cancers [12–19, 22, 32–34]. In clear cell RCC, SNHG12, as a competitive endogenous RNA, competes with miR-30a-5p to bind to downstream oncogenes RUNX2, IGF-1R and WNT2 to promote tumor cell invasiveness [19]. SNHG12, as a sponge of miR-129-5p, regulates the expression of MDM4 (a regulatory factor in p53 pathway) and p53 pathway during the development of clear cell RCC [35]. In addition, SNHG12 up-regulates CDCA3 expression by stabilizing the transcription factor SP1, thereby regulating the SNHG12/SP1/CDCA3 pathway to promote the proliferation, migration, invasion, and drug resistance of RCC [21]. SNHG12 also regulates HIF1α by competing with miR-199a-5p, thereby promoting its carcinogenic potential. However, SNHG12 deletion inhibits cell viability, anchorage-independent growth and induces apoptosis. Silencing of SNHG12 can inhibit RCC cell migration and invasion in vitro, and inhibit growth of xenograft tumors in vivo [36]. In addition, SNHG12 can up-regulate the expression of its target gene COL11A1 (collagen type XI α1 chain) through miR-200c-5p, indicating that the SNHG12/miR-200c-5p/COL11A1 axis is crucial to the progression of RCC [20].

Here, in this study, the biological function and mechanism of SNHG12 in the occurrence and development of RCC were investigated. We first found that SNHG12 was highly expressed in RCC tissues and cells. In addition, the high expression of SNHG12 was related to the tumor grade and poor prognosis of RCC patients, and the expression of SNHG12 in the tissues of informed RCC patients was related to tumor grade, TNM staging, and lymph node metastasis. To further explore the effect of SNHG12 on the biological functions of RCC cells, we knocked down SNHG12 in 786-O cells and found that SNHG12 knockdown inhibited RCC cell proliferation, migration, and invasion and HUVEC angiogenesis. A meta-analysis showed that high SNHG12 expression in a variety of tumors reduced the overall survival rate and recurrence-free survival rate of tumor patients and that the high expression of SNHG12 suggested unfavorable clinicopathological results, including larger tumors, lymph node metastasis, distant metastasis, and later clinical staging [37].

To further reveal the mechanism of high expression of SNHG12 in RCC, we searched the UCSC database and found that the promoter region of SNHG12 was enriched in H3K4me3. Next, we tested the enrichment of H3K4me3 in the promoter region of SNHG12 through ChIP and found that KMT2B up-regulated the expression of SNHG12 by mediating the modification of H3K4me3 in the promoter region of SNHG12. In addition, we predicted the possible binding sites of E2F1 in the CEP55 promoter region, and knocked down E2FF1 to explore the downstream factors of SNHG12 in RCC. We found that in RCC cells, SNHG12 overexpression promoted CEP55 expression by recruiting E2F1. Finally, we constructed a nude mouse xenograft tumor model. It was found that SNHG12 knockdown blocked the recruitment of E2F1 by SNHG12, and then down-regulated CEP55 expression, leading to the inhibition of RCC growth and angiogenesis. Our results confirmed that the SNHG12/E2F1/CEP55 axis affected RCC growth and angiogenesis.

## Conclusion

**Fig. 7:**
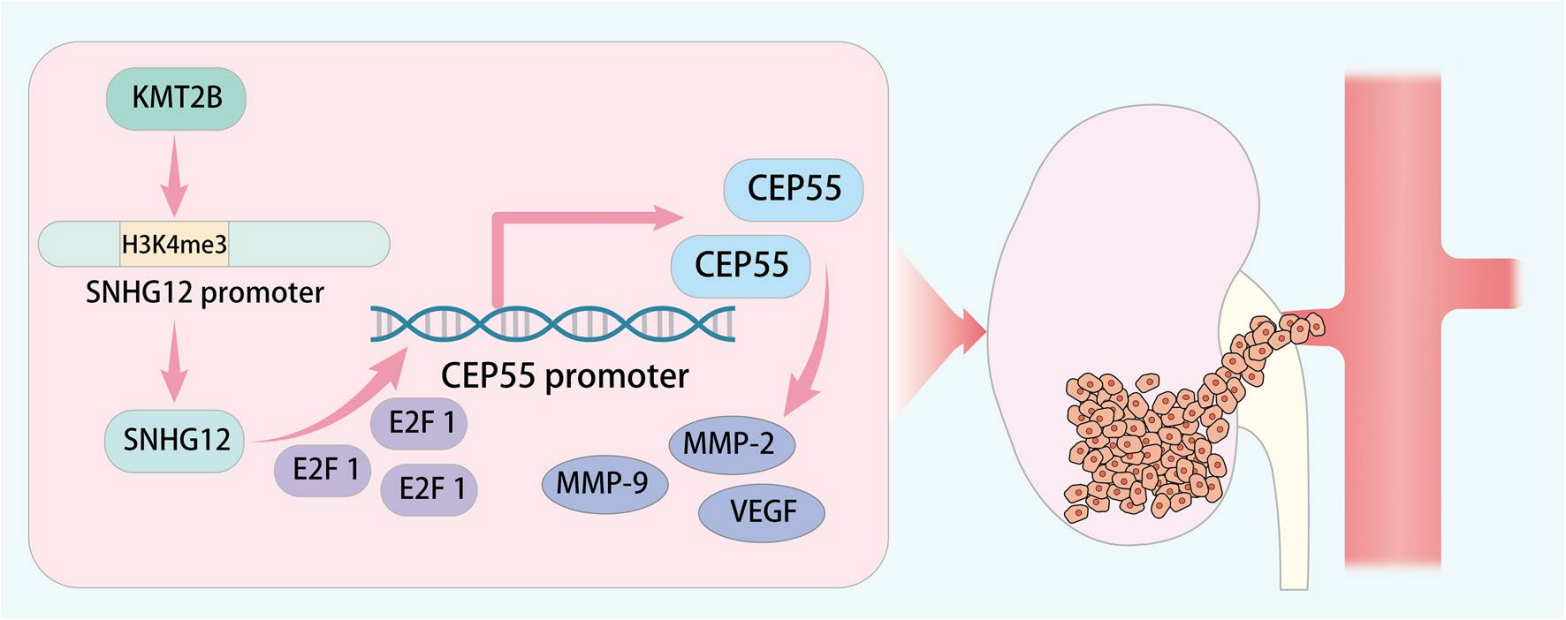
Schematic diagram of the mechanism of KMT2B in RCC. KMT2B up-regulates lncRNA SNHG12 expression through the modification of H3K4me3, which in turn recruits the transcription factor E2F1, and ultimately promotes the expression of CEP55. By this mechanism, KMT2B promotes the proliferation, migration, and invasion of RCC cells, as well as the angiogenesis of HUVECs, eventually leading to RCC growth and angiogenesis

In summary, KMT2B can up-regulate SNHG12 expression through the modification of H3K4me3 in the SNHG12 promoter region, which in turn recruits the transcription factor E2F1, and ultimately promotes expression of CEP55, and the proliferation, migration, and invasion of RCC cells, as well as the angiogenesis of HUVECs (Fig. 7). These all ultimately promote RCC growth and angiogenesis. The KMT2B/SNHG12/E2F1/CEP55 axis may enable the development of new therapeutic strategies for the treatment of RCC.

## Supplementary Information

**Additional file 1: Table S1.** shRNA sequences. **Table S2.** Primer sequences for RT-qPCR. **Table S3.** Relationship between SNHG12 expression and clinicopathological characteristics of RCC patients. **Table S4.** The transcription factors involved in the regulation of SNHG12 on CEP55 in RCC as predicted by LncMAP. **Table S5.** JASPAR analysis of the binding site of the E2F1 in the promoter region of CEP55.

## Data Availability

The datasets used and/or analysed during the current study are available from the corresponding author on reasonable request.

## Abbreviations

RCC: Renal cell carcinoma
LncRNA: LncRNA long non-coding RNA
SNHG12: Small nucleolar RNA host gene 12
E2F1: E2F transcription factor 1
CCK-8: Cell counting kit-8
CEP55: Centrosome protein 55
ChIP: Chromatin immunoprecipitation
RIP: RNA immunoprecipitation
RT-Qpcr: Real time quantitative PCR
Sh: Short hairpin RNA
oe: Overexpression
NC: Negative control
GAPDH: Glyceraldehyde-3-phosphate dehydrogenase
KMT2B: Lysine methyltransferase 2B
VEGF: Vascular endothelial growth factor
ANOVA: Analysis of Variance
HUVEC: Human umbilical vein endothelial cell
MMP: Matrix metalloproteinase
