# Supplementary material for: KMT2B promotes the growth of renal cell carcinoma via upregulation of SNHG12 expression and promotion of CEP55 transcription
Source: Cancer Cell Int. 2022 May 21;22:197. doi: 10.1186/s12935-022-02607-w (PMC9123657; doi:10.1186/s12935-022-02607-w)
Supplement: Supplementary file 1 — Additional file 1: Table S1. shRNA sequences. Table S2. Primer sequences for RT-qPCR. Table S3. Relationship between SNHG12 expression and clinicopathological characteristics of RCC patients. Table S4. The transcription factors involved in the regulation of SNHG12 on CEP55 in RCC as predicted by LncMAP. Table S5. JASPAR analysis of the binding site of the E2F1 in the promoter region of CEP55. [file 12935_2022_2607_MOESM1_ESM.docx]

**Table S1. shRNA sequences**

| shRNA | Sequence |
| --- | --- |
| sh-SNHG12-1 | 5'-GACTCTTAAGATGACAGAAGG-3' |
| sh-SNHG12-2 | 5'-GCAGATGAAGACTCTTAAGAT-3' |
| sh-SNHG12-3 | 5'-GGGTAATGACAGTGATGAAAT-3' |
| sh-KMT2B-1 | 5'-CCAGCACTATAAGTTCCGTTA-3' |
| sh-KMT2B-2 | 5'-CGCATGGATGACTTTGATGTA-3' |
| sh-KMT2B-3 | 5'-CCTGAAGAATATCCGGCAGTT-3' |
| sh-E2F1-1 | 5'-ACATCACCAACGTCCTTGAG-3' |
| sh-E2F1-2 | 5'-CGCTATGAGACCTCACTGAAT-3' |
| sh-E2F1-3 | 5'-TAAGAGCAAACAAGGCCCGAT-3' |

Note: sh-, shRNA, short hairpin RNA; SNHG12, small nucleolar RNA host gene 12; E2F1, E2F transcription factor 1.

**Table S2. Primer sequences for RT-qPCR**

| Gene | Sequence |
| --- | --- |
| KMT2B (human) | Forward: 5'-TGACAAGTGTGAATCCCGTGAAG-3' |
|  | Reverse: 5'-AACCATTTCATCCGTTGTTACGAAG-3' |
| SNHG12 (human) | Forward: 5'-TCTGGTGATCGAGGACTTCC-3' |
|  | Reverse: 5'-ACCTCCTCAGTATCACACACT-3' |
| E2F1 (human) | Forward: 5'-ATGTTTTCCTGTGCCCTGAG-3' |
|  | Reverse: 5'-ATCTGTGGTGAGGGATGAGG-3' |
| CEP55 (human) | Forward: 5'-AGTAAGTGGGGATCGAAGCCT-3' |
|  | Reverse: 5'-CTCAAGGACTCGAATTTTCTCCA-3' |
| GAPDH (human) | Forward: 5'-GGAGCGAGATCCCTCCAAAAT-3' |
|  | Reverse: 5'-GGCTGTTGTCATACTTCTCATGG-3' |

Note: KMT2B, lysine methyltransferase 2B; SNHG12, small nucleolar RNA host gene 12; E2F1, E2F transcription factor 1; CEP55, centrosome protein 55; GAPDH, glyceraldehyde-3-phosphate dehydrogenase.

**Table S3. Relationship between SNHG12 expression and clinicopathological characteristics of RCC patients.**

| **Variable** | **N** | **SNHG12 expression** | | ***P* value** |
| --- | --- | --- | --- | --- |
|  |  | High | Low |  |
| Age (year) | | | | 0.765 |
| < 60 | 27 | 14 | 13 |  |
| ≥ 60 | 19 | 9 | 10 |  |
| Gender | | | | 0.536 |
| Male | 30 | 16 | 14 |  |
| Female | 16 | 7 | 9 |  |
| TNM stage | | | | 0.047 |
| I-II | 33 | 13 | 20 |  |
| III-IV | 13 | 10 | 3 |  |
| Fuhrman grade | | | | 0.006 |
| G1-2 | 28 | 9 | 19 |  |
| G3 | 18 | 14 | 4 |  |
| Lymph node metastasis | | | | 0.035 |
| Yes | 11 | 9 | 2 |  |
| No | 35 | 14 | 21 |  |

Note: The data were analyzed by Chi-square test. However, if T < 5, the data were further analyzed by Fisher's method.

**Table S4.** The transcription factors involved in the regulation of SNHG12 on CEP55 in RCC as predicted by LncMAP.

| LncRNA Symbol | TF Symbol | Gene Symbol |
| --- | --- | --- |
| SNHG12 | E2F1 | CEP55 |
| SNHG12 | PBX3 | CEP55 |

**Table S5.** JASPAR analysis of the binding site of the E2F1 in the promoter region of CEP55.

| Matrix ID | Name | Score | Relative score | Sequence ID | Start | End | Strand | Predicted sequence |
| --- | --- | --- | --- | --- | --- | --- | --- | --- |
| MA0024.1 | E2F1 | 7.17655 | 0.813289007993 | CEP55 | 292 | 299 | + | tttgcccc |
| MA0024.1 | E2F1 | 7.17655 | 0.813289007993 | CEP55 | 1091 | 1098 | - | ttagccgc |
| MA0024.1 | E2F1 | 6.81352 | 0.800797328565 | CEP55 | 836 | 843 | - | tttcccga |
